# Carbon source utilization regulates biofilm formation and plant-beneficial interactions of *Pseudomonas ogarae* F113

**DOI:** 10.1016/j.isci.2025.113639

**Published:** 2025-09-24

**Authors:** Théophile Franzino, Hasna Boubakri, Lisa Merlin, Amandine M’Sakni, Michel Droux, Florian Mermillod-Blondin, Yvan Moënne-Loccoz, Mohammed Bendahmane, Judit Szécsi, Feth el Zahar Haichar

**Affiliations:** 1 INSA-Lyon, Université Claude Bernard Lyon 1, CNRS, UMR5240 Microbiologie, Adaptation, Pathogénie, 10 rue Raphaël Dubois, 69622 Villeurbanne, France; 2 Laboratoire Reproduction et Développement des Plantes, ENS de Lyon, Université Claude Bernard Lyon 1, CNRS, INRAE, 69364 Lyon, France; 3 Université Claude Bernard Lyon 1, UMR CNRS 5557 Laboratoire d'Ecologie Microbienne, INRAE, VetAgro Sup, 69622 Villeurbanne, France; 4 Université Claude Bernard Lyon 1, CNRS, ENTPE, UMR5023 Écologie des Hydrosystèmes Naturels et Anthropisés (LEHNA), 69622 Villeurbanne, France; 5 Institut Universitaire de France, 75005 Paris, France

**Keywords:** Microbiology, Plant Biology, Interaction of plants with organisms

## Abstract

Root exudate determines the gene expression of root associated bacteria, but the underlying mechanisms are poorly understood. We tested the hypothesis that carbon sources within root exudates regulate interactions of beneficial bacteria with the plant via carbon catabolite repression (CCR). Mutants in CCR genes were constructed in *Pseudomonas ogarae* F113, and rhizospheric traits were studied. *P. ogarae* F113 displays reverse CCR. The *Δcrc* mutant produced more biofilm than the wild-type strain, and its swimming was carbon source dependent. On roots, the bacterial expression of *nirS, crcY,* and *crcZ* was higher in the *Δcrc* mutant. Auxin expression (but not jasmonate signaling) in *Arabidopsis thaliana* was reduced upon inoculation by *Δcrc* or *ΔcbrB* mutants when compared with the wild-type strain. Our findings show that reverse CCR regulates the communication between *P. ogarae* F113 and *A. thaliana*, which sheds further light on the significance of root exudates for the functioning of plant-beneficial bacteria on roots.

## Highlights

• Carbon Catabolite Repression (CCR) regulates *Pseudomonas ogarae* F113 traits
• *P. ogarae* F113 displayed a reverse CCR mechanism
• Reverse CCR mechanism regulates biofilm formation and swimming
• *Arabidopsis* auxin metabolism was affected by *Pseudomonas* CCR mutants

## Graphical abstract

**Figure:**
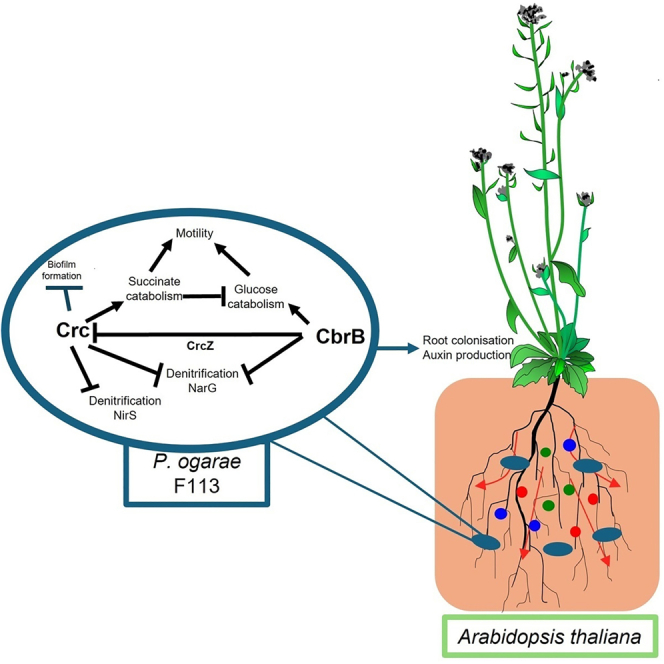

## Introduction

The plant microbiota provides key benefits by enhancing growth, stress resistance, and health of plants^1^^,^^2^^,^^3^ and it is of paramount importance to understand the mechanisms that drive the build-up of the plant holobiont. From an autecological viewpoint, this requires developing our basic knowledge on the properties needed by individual plant-beneficial microorganisms to interact with their plant partner.^4^^,^^5^^,^^6^

Bacteria successfully colonize plant roots when they possess specific traits referred to as rhizosphere competence^7^ such as root exudate catabolism, respiration flexibility, motility and chemotaxis, biofilm formation, or biosynthesis of secondary metabolites.^8^ Among respiration processes, denitrification is one of the widely expressed functions in the rhizosphere of plants, and the genes involved in this process have been shown to play a role in the fitness of bacteria to grow anaerobically as well as in rhizospheric competence.^7^ The implementation of these microbial traits requires the optimal regulation of gene expression in the rhizosphere, as environmental conditions experienced by microorganisms on roots can vary when considering (i) rhizosphere heterogeneity, as well as (ii) differences in rhizosphere effects produced by distinct plant species.^3^^,^^6^^,^^9^ These effects are largely related to the composition of exudates released by roots.^10^^,^^11^

A key aspect of the environmental conditions prevailing in the rhizosphere is the availability of organic compounds released by roots (rhizodeposits). Consequently, metabolic versatility is a key asset for rhizobacteria on roots, allowing them to use as carbon and energy sources many different compounds present in root exudates. This facilitates their colonization of different rhizosphere microhabitats and their adaptation to changing environmental conditions on roots. However, it is poorly understood how the presence of particular organic substrates in root exudates regulates gene expression in root-associated bacteria and their plant-beneficial effects. Bacteria in different natural or anthropized ecosystems can either co-metabolize different carbon sources or preferentially assimilate a specific class of compounds that provide the most efficient growth. Simultaneously, along with the preferential assimilation, bacteria finely trigger the inhibition of numerous functions, including those involved in the catabolism of non-preferred compounds. These regulatory processes, allowing for the selection of preferred carbon sources, have been named Carbon Catabolite Repression (CCR).^12^ Bacteria, such as *Enteroccocus* and many Firmicutes genera, display the classical CCR with a preference for sugar over organic acids, while certain *Pseudomonas* are known to be characterized by the reverse CCR (revCCR), such as *P. aeruginosa* or *P. putida*, which use organic acids such as succinate as a preferred carbon source.^8^^,^^13^

Among rhizobacteria, plant growth-promoting rhizobacteria (PGPR) exert beneficial effects on plant growth and development through direct and indirect mechanisms.^11^ It is known that 5–10% of all bacterial genes are subject to CCR,^12^ and it can be expected that CCR regulations are important in a rhizosphere setting. However, the effect of CCR on PGPR bacterial functions is poorly characterized.

In this article, we tested the hypothesis that CCR regulates nutrient transformation and the interaction of plant-beneficial bacteria with plants, in response to the presence of a particular organic substrate known to occur in root exudates. To this end, we examined the implication of the carbon catabolite repression mechanism on (i) bacterial nutrient transformation aerobically and anaerobically (denitrification) and (ii) bacterial rhizospheric traits during *Arabidopsis thaliana-Pseudomonas ogarae* F113 interaction. *P. ogarae* F113 is a well-known PGPR isolated from sugar beet and capable of colonizing a wide range of plant roots^6^^,^^14^^,^^15^ due to its rhizospheric traits such as denitrification,^4^^,^^16^ biofilm formation,^9^ and swimming.^17^ Thanks to those bacterial traits, this PGPR could improve plant growth by stimulating root growth, nutrient, and water uptake, or active plant immunity to defend against pathogen attacks, as observed for other PGPRs.^3^^,^^18^ The Arabidopsis – PGPR system was shown to be very useful in order to study beneficial effects and discover novel underlying molecular mechanisms during microbial root colonisation.^19^

Here, we revealed the implication of revCCR and its major regulators, Crc (a global regulator of catabolite repression) and CbrB (the response regulator of the CbrAB two component system), on the *in vitro* growth of *P. ogarae* F113 in aerobic and anaerobic conditions. Moreover, we evidenced that the expression of denitrification genes is regulated by revCCR. Next, we investigated the effect of the revCCR and its regulators on diverse bacterial traits. More precisely, we showed that revCCR positively affects bacterial swimming ability and the capacity to induce auxin signaling in plant cells, and these processes implicate both Crc and CbrB. On the contrary, revCCR seems to negatively affect biofilm formation in a Crc dependent manner. All these data demonstrate the importance of revCCR and thus of the carbon source quality of root exudates in regulating bacterial traits important for rhizosphere adaptation and interaction with the plant.

## Results

### *P. ogarae* F113 presents a reverse carbon catabolite repression mechanism where crc and CbrB act as master regulators under aerobic conditions

**Figure 1:**
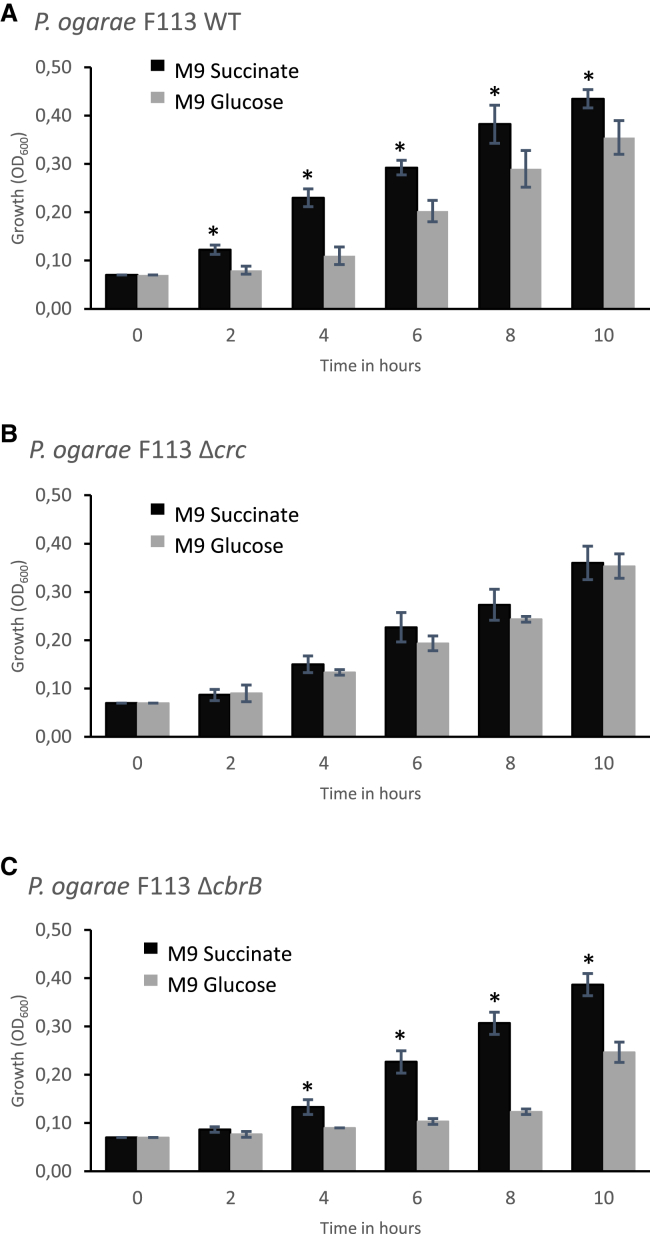
Kinetic growth in aerobic conditions of *Pseudomonas ogarae* F113 WT and its mutants deleted in *Crc* (*Δcrc*) or *CbrB* (*ΔcbrB*) genes (A–C) Histogram representation of growth of (A) F113 wild type, (B) F113 *Δcrc,* and (C) F113 *ΔcbrB*. Cells were grown in M9 minimal medium supplemented with 40 mM of carbon either from succinate (black bar) or glucose (gray bar) as sole carbon sources in aerobic conditions and OD 600 nm were measured during time course in hours. Error bars correspond to the standard deviation of three biological replicates. Stars indicate a *p*-value <0.05 (Mann-Whitney’s U-test).

To determine which type of CCR is expressed by root-colonizing *P. ogarae* F113, the strain was grown aerobically in M9 medium supplemented with succinate or glucose (two types of compounds typically prominent in root exudates) as the only source of carbon in aerobic conditions. Wild-type strain showed a 2 h delayed onset of growth in the presence of glucose as a C source compared with the succinate condition (Figure 1A). In addition, the growth rate μ between 2 and 4 h of growth was 0.22 (±0.09) h^−1^ with glucose vs. as much as 0.45 (±0.02) h^−1^ with succinate. These data show that *P. ogarae* F113 does indeed prefer succinate over glucose as a C source, and hence confirm a revCCR mechanism. Then, to assess the role of the major CCR actors Crc and CbrB, we constructed deletion mutants for *crc* and *cbrB* (F113 *Δcrc* and F113 *ΔcbrB,* respectively) in *P. ogarae* F113 and tested their growth in the same conditions as above. No difference in growth was observed between the two C sources for the *Δcrc* mutant (Figure 1B), pointing to the importance of Crc in substrate ranking. With succinate, the *ΔcbrB* mutant grew similarly to the wild-type strain (μ = 0.38 (±0.04) h^−1^ between 4 and 6 h of growth), while its growth was delayed during the first 8 h under glucose conditions (Figure 1C) and was lower (μ = 0.09 (±0.04) h^−1^) in comparison with the wild-type strain. This indicated that F113 *ΔcbrB* has a longer adaptation time to use sugar as a carbon source, and suggests that CbrB plays a role in the relief of catabolic repression. The complementation of the two mutants by the expression of respectively *crc* or *cbrB*, each under the control of its native promoter, successfully restored the preference of succinate over glucose as a sole carbon source, as observed with the wild-type strain (see Figure S1).

In summary, *P. ogarae* F113 displayed a preference for the organic acid succinate over the sugar glucose for growth in aerobic conditions (i.e., revCCR regulation), and this catabolic repression required functional *crc* and *cbrB* genes.

### Effect of carbon source on growth and N transformations by *P. ogarae* F113 under anaerobic conditions

**Figure 2:**
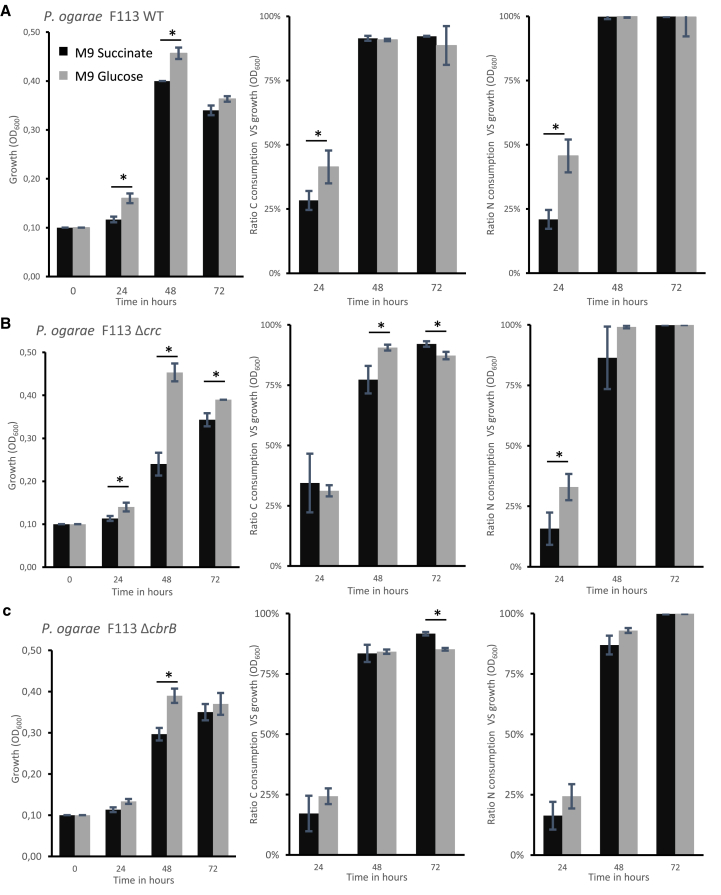
Kinetic growth in anaerobic conditions of *Pseudomonas ogarae* F113 WT and its mutants Δ*crc* and Δ*cbrB* (A–C) Histogram representation of growth and ratio of carbon or nitrogen sources consumption reported by the growth for F113 (A), F113 *Δcrc* (B) and F113 *ΔcbrB* (C) under anaerobic conditions. Cells were grown in M9 minimal medium supplemented with 20 mM of KNO_3_ and with 40 mM of carbon either from succinate (black bar) or glucose (gray bar) as the sole carbon source under anaerobic conditions. Error bars correspond to the standard deviation of three biological replicates. Stars indicate a *p*-value <0.05 (Mann-Whitney’s U-test).

When grown anaerobically in M9 supplemented with KNO_3_ and glucose, *P. ogarae* F113 grew better at 24 h and 48 h and displayed a better carbon and nitrate consumption (by a factor of 1.5 and 2.0, respectively) at 24 h compared with succinate (Figure 2A), meaning that revCCR exerts a negative effect on denitrification and growth under these conditions. Like the wild-type strain, the mutant F113 *Δcrc* grew better with glucose than succinate (almost 2-fold at 48 h; Figures 2A and 2B), with a tendency for nitrate consumption. In the mutant F113 *ΔcbrB*, preferential use of glucose was delayed compared with the wild-type strain and the F113 *Δcrc* mutant strain, and growth was only observed from 48h onwards (Figure 2C). In addition, compared with the wild-type strain, growth of F113 *ΔcbrB* was negatively affected, both with succinate and glucose, suggesting that CbrB is important for optimal growth under denitrification conditions. It is interesting to note that the N source was totally consumed by all three strains during the 48 h of growth (Figure 2). In summary, under anaerobic conditions, sugar was preferentially used over organic acid for growth and nitrate utilization. In the mutants F113 *ΔcbrB* and F113 *Δcrc*, anaerobic growth on succinate is further impacted, pointing out that revCCR contributes to growth when the preferred C source is available.

### Reverse carbon catabolite repression mediated control of bacterial fitness *in vitro*

**Figure 3:**
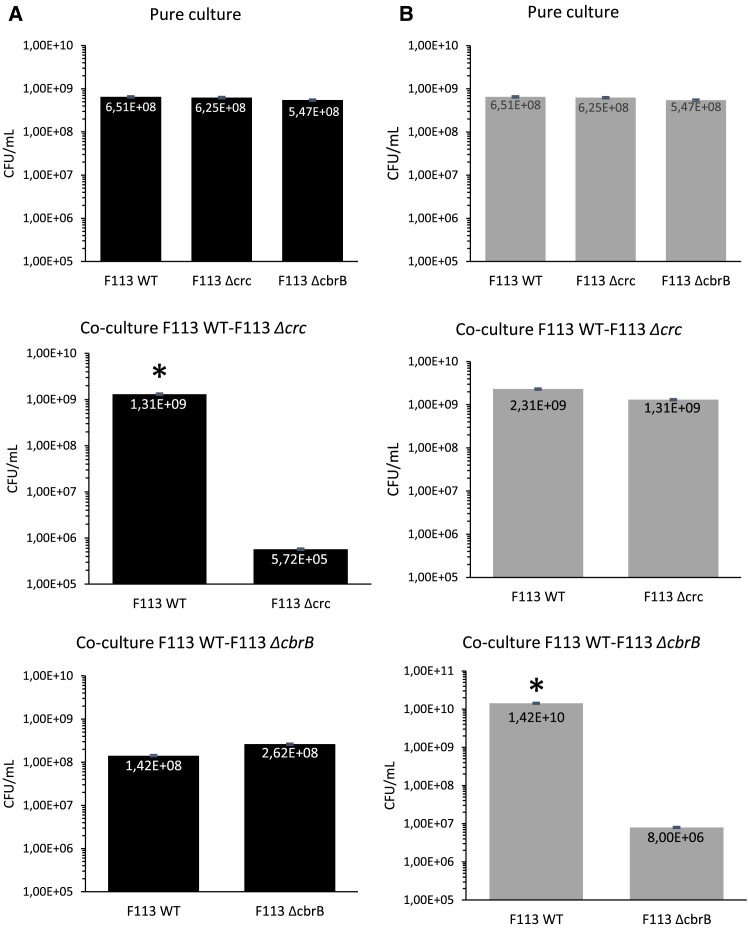
Competition assays between F113 WT and F113 Δcrc or F113 ΔcbrB strains *in vitro* with either succinate or glucose as sole carbon source (A and B) The numerations of cells were performed using strains colored with fluorescent proteins (RED and GFP proteins). The two combinations of colored strains were tested, and no difference was observed. Growth of F113 WT and mutant strains in pure and co-culture during *in vitro* assays with Succinate (A) or Glucose (B) as sole carbon source. The cell concentrations were determined for the initial inoculum and 24 h after inoculation. Error bars correspond to standard deviation (*n* = 3). Stars indicate a *p*-value <0.05 (Mann-Whitney’s U-test).

In M9 supplemented with either succinate or glucose, *P. ogarae* F113, F113 *Δcrc* and F113 *ΔcbrB* inoculated singly at approximately 1.5 × 10^8^ CFU mL^−1^ reached similar levels at 24 h, respectively 6.5 × 10^8^ CFU mL^−1^, 6.3 x 10^8^ CFU mL^−1^, and 5.5 x 10^8^ CFU mL^−1^ (Figure 3). There was no difference in growth with succinate or glucose (Figures 3A and 3B).

Following co-culture of *P. ogarae* F113 and F113 *Δcrc* in M9 containing succinate, strain F113 reached 1.3 × 10^9^ CFU mL^−1^ while the mutant F113 *Δcrc* decreased to 5.7 × 10^5^ CFU mL^−1^ (Figure 3A). In the presence of glucose, the wild-type F113 and F113 *Δcrc* reached 2.3 × 10^9^ CFU mL^−1^ and 1.3 × 10^9^ CFU mL^−1^, respectively (Figure 3B). When cultured together in presence of succinate, *P. ogarae* F113 wild-type reached 1.4 × 10^8^ CFU mL^−1^ and reach 1.4 x 10^10^ CFU mL^−1^ on glucose (Figures 3A and 3B), strain F113 *ΔcbrB* was recovered at 1.42 × 10^8^ CFU mL^−1^ in presence of succinate but decreased to 8.0 × 10^6^ CFU mL^−1^ in presence of glucose (Figures 3A and 3B).

In summary, the mutants F113 *Δcrc* and F113 *ΔcbrB* grew as well as *P. ogarae* F113, when alone, using either succinate or glucose. In competition with the wild-type strain, however, growth of F113 *Δcrc* was impaired on succinate, and that of F113 *ΔcbrB* on glucose, pointing to the importance of Crc and CbrB in C metabolism and competition for nutrient use.

### Reverse carbon catabolite repression mediated control of key bacterial traits for rhizosphere colonization

**Figure 4:**
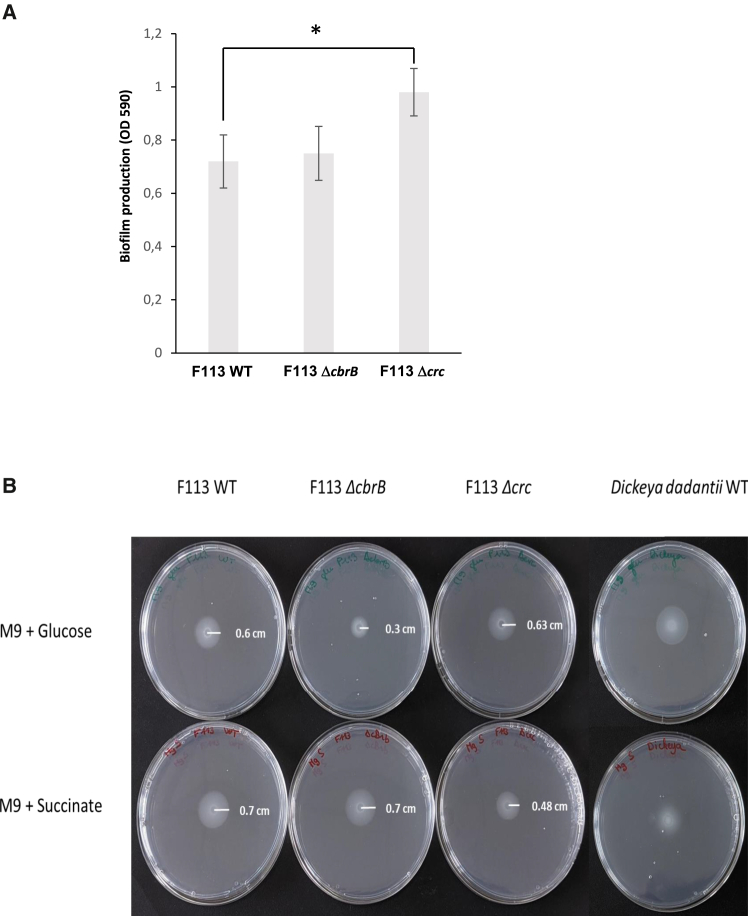
Impact of revCCR on different rhizospheric traits (A) Visualization and quantification of biofilm formation in *P. ogarae* F113 WT, *ΔcbrB* and *Δcrc* mutant strains. Biofilm adhered were stained with crystal violet and the formed biofilm was quantified using a spectrophotometer at OD_590nm_. Error bars correspond to the standard deviation of three biological replicates. Stars indicate a *p*-value <0.05. (B) Visualization and quantification of swimming motility in *P. ogarae* F113 WT and in *ΔcbrB* and *Δcrc* mutant strains. Strains were grown on soft M9 medium supplemented with succinate (M9S) or glucose (M9G) as sole carbon source. The experiment was performed in quadriplicates and image of representative plates are shown.

When testing the influence of revCCR on biofilm formation, a significant increase (×1.5) in biofilm formation was found for the *Δcrc* mutant compared with the wild-type strain at 24 h *in vitro* (Figure 4A), suggesting that Crc negatively regulates biofilm formation in *P. ogarae* F113. In contrast, no difference with the wild-type strain was observed for the Δ*cbrB* mutant.

The swimming behavior of bacteria is influenced by nutrient availability (Yan et al., 2019). Here, the swimming ability of *P. ogarae* F113 was similar with glucose (swimming radius sr = 0.60 ± 0.08 cm) and succinate (sr = 0.70 ± 0.00 cm) (Figure 4B). In comparison with the wild-type strain, the swimming ability of the *Δcrc* mutant was lower on succinate (sr = 0.48 ± 0.05 cm) but not glucose (sr = 0.63 ± 0.00 cm), and that of the *ΔcbrB* mutant was lower on glucose (sr = 0.30 ± 0.00 cm) but not succinate (sr = 0.70 ± 0.00 cm).

In summary, revCCR regulates both biofilm formation and swimming. Crc is needed to maintain optimal swimming in the presence of the preferred C source succinate, and CbrB has a role as a revCCR reliever regulator in the presence of glucose.

### Expression of reverse carbon catabolite repression genes and denitrification genes during root colonization by *P. ogarae* F113

**Figure 5:**
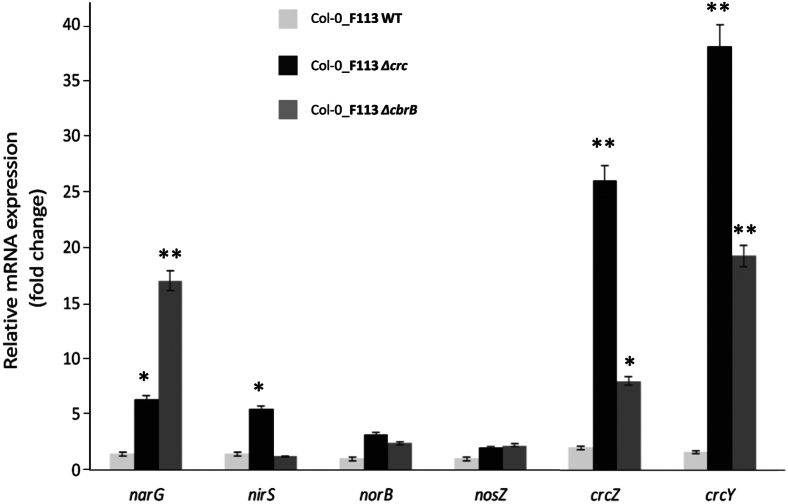
Expression profile of genes involved in denitrification and revCCR mechanism during the root colonization of *Arabidopsis thaliana* plant Gene expression profile was analyzed after 3 days post inoculation. Effect of Δ*crc* and Δ*cbrB* F113 mutants on the expression of *narG*, *nirS*, *norB*, *nosZ*, *crcZ,* and *crcY* after their inoculation in *A. thaliana WT line.* Fold change in gene expression was normalized using *rpoD* as housekeeping bacterial gene and calculated using the ΔΔCt method. Quantitative real-time PCR was performed in triplicate, and the data presented are the mean of relative change, and the error bar represents standard deviation. ∗, significant difference *p* < 0.05; ∗∗, significant difference *p* < 0.01.

When assessing the expression profile of bacterial genes implicated in revCCR (*crcZ, crcY*) and denitrification (*narG, nirS, norB, nosZ*) pathways^20^ during the root colonization of *A. thaliana*, we observed a 5-fold and 17-fold increase of *narG* expression in the mutants F113 *Δcrc* and F113 *ΔcbrB*, respectively, compared with the wild-type strain (Figure 5). *nirS* expression was 5-fold higher in F113 *Δcrc* mutant but was not affected in F113 *ΔcbrB*, whereas the expression of *norB* and nosZ did not differ significantly between the three strains. Interestingly, *crcY* and *crcZ* were overexpressed in each mutant compared with the wild-type strain.

In summary, *P. ogarae* F113 *Δcrc* and F113 *ΔcbrB* colonizing roots exhibited enhanced expression of genes involved in nitrate or nitrite reduction, meaning that early denitrification steps are negatively regulated by revCCR. In addition, the expression patterns of the sRNA genes *crcY* and *crcZ* suggest that they play a role in revCCR regulation *in planta*.

### Reverse carbon catabolite repression mediated control of rhizosphere colonization

**Figure 6:**
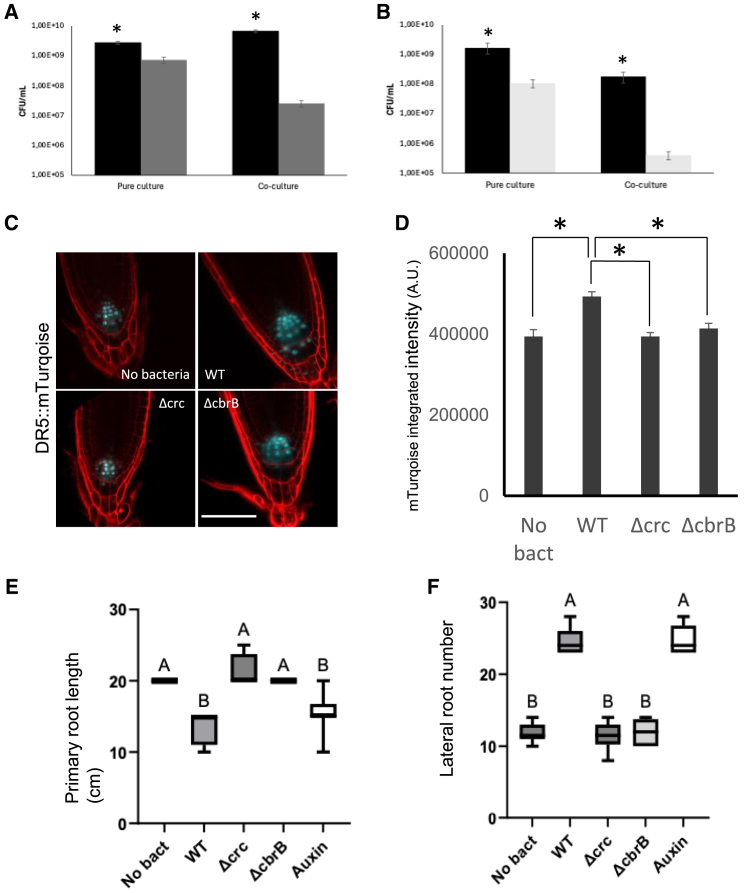
Effect of rev CCR on root colonization and competition assays (A, B), plant auxin signaling (C-D) and root length and density (E and F) (A and B) Roots colonization and competition assays between F113 WT (black bar) and F113 Δ*crc* (gray bar) or F113 Δ*cbrB* (light gray bar) strains *in planta. A. thaliana* roots were inoculated with a pure culture or a mixture of F113 WT and F113 Δ*crc* (A) or F113 WT and Δ*cbrB* (B) at a 1:1 ratio. (C and D) The proportion of each strain recovered from the *A. thaliana* roots was determined after 24 h of inoculation. Error bars correspond to standard deviation (*n* = 4). Effect of revCCR on plant auxin signaling (C and D). *A. thaliana* roots were inoculated with F113 WT, F113 Δ*crc* or F113 Δ*cbrB* and their effect on auxin signaling was determined 24h after inoculation. (C) Representative images of roots harboring the DR5::mTurquoise auxin signaling marker after inoculation with F113 WT or mutant strains. Bar represents 50 μm. (D) Quantification of mTurquoise integrated intensity. Error bars correspond to standard error (*n* = 20). Stars indicate a *p*-value <0.01 (Student’s t test). (E and F) *A. thaliana* roots were inoculated with F113 WT, F113 Δ*crc,* or F113 Δ*cbrB* and primary root length and secondary root density were determined 7 days after inoculation. (E) Quantification of primary root length and lateral root density (F). Error bars correspond to standard error (*n* = 8). Different letters indicate statistically significant differences (ANOVA, *p* < 0.0001).

Competition assays were run *in planta*, in which *P. ogarae* F113 WT, mutants F113*Δcrc* or F113*ΔcbrB* were inoculated on *A. thaliana* roots alone or co-inoculated with F113 WT/F113 *Δcrc* or F113 WT/F113 *ΔcbrB* in a 1:1 proportion. During the first day, F113*Δcrc* and F113*ΔcbrB,* inoculated alone, grew significantly less than the WT strain. In addition, in both co-inoculated conditions, the wild-type strain grew better than mutant strains (Figures 6A and 6B), suggesting that both CbrB and Crc are important for *P. ogarae* F113 root colonization and competition *in planta.*

### Reverse carbon catabolite repression mediated control of bacterial effects on plant hormone signaling

PGPR bacteria, in general, are known to affect phytohormone signaling in plants. Their effect on auxin and jasmonate signaling are well characterized,^21^^,^^22^ but the link between revCCR and plant hormone signaling is yet to be uncovered. To determine whether the revCCR of *P. ogarae* F113 affects auxin homeostasis in *A. thaliana* during root colonization, wild-type and mutant strains were inoculated on roots of Col-0 containing the *DR5::mTurquoise* auxin sensor. In the presence of the F113 WT strain, auxin maxima in the roots 24 h after inoculation were higher than in the absence of bacteria (Figures 6C and 6D), as expected. In contrast, inoculation with either F113 *Δcrc* or F113 *ΔcbrB* had no effect on *DR5::mTurquoise* fluorescence intensity, which remained similar to that in non-inoculated roots (Figures 6C and 6D). Moreover, increased auxin concentration affects primary root length and lateral root (LR) density (Figures 6E and 6F). In roots infected with the F113 WT strain, we observed shorter primary roots and increased LR density, in agreement with the increased auxin signaling (Figures 6E and 6F). LR density in these plants was similar to that observed after exogenous auxin treatment (Figures 6E and 6F). On the contrary, LR density upon F113 *Δcrc* or F113 *ΔcbrB* inoculation was comparable to that observed in non-treated roots (Figures 6E and 6F). These results together point toward a major role of revCCR in auxin signaling and root architecture modification.

In parallel, we also investigated the role of revCCR in jasmonate signaling in *A. thaliana* using a *p35S::JAZ1-GUS* construct. Inoculation with *P. ogarae* F113, F113 *Δcrc,* or F113 *ΔcbrB* resulted in a similar decrease in GUS activity (see Figure S2), indicating that *P. ogarae* induced a plant defense response, but revCCR does not play a role in this process.

In summary, data pointed to a significant role of revCCR in modulating auxin accumulation and root architecture during bacteria-plant interactions, and this process implicates both Crc and CbrB. On the contrary, revCCR has no effect on jasmonate signaling as demonstrated by similar levels of JA signaling upon the inoculation of *P. ogarae* F113 WT or mutants on roots.

## Discussion

### Root exudate-responsive reverse carbon catabolite repression regulates aerobic and anaerobic growth in *P. ogarae* F113

The revCCR mechanism is well described in *P. aeruginosa* PAO1, where Crc and CbrB act as main regulators.^8^^,^^23^ Metabolic pathways of non-preferred carbon sources, such as sugar compounds, are under the repression of revCCR and thus *P. aeruginosa* PAO1 presents a reduced growth with glucose as the sole carbon source compared to succinate, which is the preferred carbon source.^24^^,^^25^ Here, we observed the same growth profile for *P. ogarae* F113 under aerobic conditions, confirming the revCCR regulation for this strain. In addition, our data show that in the absence of Crc, *P. ogarae* F113 no longer expresses revCCR and was able to grow in the presence of glucose as the sole carbon source without any delay, opposite to what was observed with the F113 WT strain, demonstrating the role of Crc in carbon catabolite repression. This result is also in agreement with the observation of Molina and colleagues (2019)^26^, where *Pseudomonas putida* KT2440 Δ*crc* mutant presents a higher glucose consumption rate during the early stages of growth, concomitant with a high growth level. Moreover, in the presence of glucose as the sole carbon source, the F113 Δ*cbrB* mutant was still able to grow but with a greater delay than the wild-type strain, which is in agreement with Nishijyo et al. (2001)^27^ where PAO1 Δ*cbrB* mutant expressed a growth defect in the presence of glucose as a sole carbon source. This result demonstrates the regulatory role of CbrB in the relief of the catabolite repression. Indeed, in *Pseudomonas* species*,* it is well known that CbrB is implicated in the expression of the sRNA CrcZ (which sequesters Crc) in order to remove catabolic repression.^24^ In addition to CrcZ, other sRNAs participate in the removal of revCCR repression, such as CrcY and CrcX in *P. putida* KT2440 and *Pseudomonas syringae* pv. *tomato* DC3000, respectively.^28^^,^^29^ BLAST analysis of the *P. ogarae* F113 genome revealed the presence of *crcY* in addition to *crcZ.*^8^ CrcY is likely to be redundant with CrcZ,^30^ and it was shown in *P. putida* KT2440 that the transcription of *crcY* seems to be partially regulated by CbrB. In the absence of CbrB, *P. ogarae* F113 mutant grows in the presence of non-preferred C after the latency phase, suggesting that CrcY might take the lead over CrcZ in the relief of the revCCR mechanism, and therefore other regulatory systems independent of CbrB might interplay in the transcription of CrcY in order to relieve the repression.

In our study, we confirm the role of revCCR in *P. ogarae* F113 growth under aerobic conditions,^8^ but to our knowledge, no study has explored the role of revCCR during *P. ogarae* F113 anaerobic growth. Many bacteria can use nitrate (NO_3_^−^) as a terminal electron acceptor during the denitrification process, allowing them to grow under anoxic conditions.^7^ Under anoxic conditions, our results show a better growth of F113 strain associated with a better C and NO_3_^−^ consumption in the presence of a non-preferred C source rather than a preferred C source. This result suggests that revCCR inhibits the use of NO_3_^−^ as the terminal electron acceptor under anaerobic conditions and hence evidences the role of revCCR in denitrification regulation. Other regulating systems can also participate in the regulation of denitrification. Indeed, Pusic and colleagues (2016)^31^ suggested that *crcZ* might be regulated by the oxygen sensor ANR (FNR-like proteins), since a putative ANR binding motif (TTGAT[N_4_]ATCAA) was found in the promoter region of *crcZ* in *P. aeruginosa* PAO1. In *P. ogarae* F113, we found the same putative ANR site but, surprisingly, not in the promoter region of *crcZ* but only in the promoter region of *crcY* (data not shown). This indicates that in *P. ogarae* F113, CrcY might be more effective in relieving the repression during anaerobic growth than CrcZ. Thus, it seems important to take these other denitrification regulatory systems into account in order to gain a better understanding of denitrification regulation.

### Reverse carbon catabolite repression regulators control denitrification genes and CrcZ, CrcY sRNA expression *in planta*

CrcZ and CrcY are two small ncRNAs under the control of revCCR regulators that contribute to the relief of catabolic repression by sequestering Crc.^8^ Our hypothesis is that if revCCR, and therefore Crc, represses denitrification, the expression of ncRNAs will be associated with overexpression of denitrification genes. *P. ogarae* F113 is a denitrifying bacterium that possesses the four enzymes (NarG, NirS, NorB, and NosZ) required for progressively reducing nitrate into dinitrogen.^17^

The upregulation of *narG* and *nirS* in the absence of Crc confirms that Crc negatively affects nitrate reduction (Figure 5). This indicates that revCCR inhibits the denitrification process, as shown in our previous results (Figure 2). The overexpression of *narG* in the absence of CbrB could be explained by the disequilibrium of C/N balance held by CbrAB and NtrBC (a two-component system that enables the assimilation of nitrogen sources) systems.^30^ For sRNA, the strong induction of *crcZ* and *crcY* in the *Δcrc* mutant compared to the *ΔcbrB* might be due to the fact that CbrB controls the expression of these sRNAs, as already demonstrated in other *Pseudomonas* species.^28^ In addition, our observation that the expression is not totally repressed in the absence of CbrB suggests the involvement of another regulator in *crcZ* and *crcY* expression, such as ANR, discussed previously, or NarXL, a two-component system (TCS) which controls *narG* expression in *P. aeruginosa* PAO1 in the presence of nitrate.^32^ We also show that *crcY* is more expressed than *crcZ*, which could be explained by the potential activation of *crcY* by the ANR regulator under anaerobic conditions, as suggested by Pusic et al. (2016).

### Reverse carbon catabolite repression mechanism is involved in *P. ogarae* F113 rhizospheric traits

Franzino et al. (2022)^8^ underlined that CCR is an important trait involved in plant-microbe interactions. In order to colonize roots efficiently, bacteria possess a set of rhizospheric traits such as motility or the ability to form biofilm. A mutation in genes implicated in biofilm formation or motility in *P. putida* resulted in a decrease in its ability to colonize seeds and roots.^33^ In our study, F113 *Δcrc* mutant produced more biofilm on abiotic surfaces than the WT strain, which is in agreement with Chakravarthy et al. (2017)^34^, who showed that *P. syringae* pv. *tomato* DC3000 *Δcrc* mutant displayed increased biofilm formation. However, the ability of F113 *Δcrc* mutant to produce more biofilm on abiotic surfaces does not necessarily mirror its ability to efficiently colonize plant roots, as F113 *Δcrc* mutant showed significantly less capacity to colonize root tissues. Then we observed a significantly reduced swimming ability in the presence of glucose for the *ΔcbrB* mutant strain. In agreement with our findings, Sivakumar et al. (2021)^35^ showed that *P. aeruginosa* PGPR2 *ΔcbrB* mutant was impaired in swimming ability in the presence of sugar as a C source. In their study, the authors showed that a PGPR2 *ΔcbrB* mutant was depleted in flagella production, which is crucial for root colonization, and explained the observed decrease in corn root colonization.^35^ F113 *ΔcbrB* mutant is less competitive in colonizing *A. thaliana* plants during plant competition assays, which can be explained by its impaired swimming trait. These results evidenced the role of CbrB in root colonization and highlight the important role of the revCCR mechanisms during plant-bacteria interaction.

An important part of PGPR's beneficial action on plants is mediated by phytohormones that participate both in inducing plant growth and in reinforcing plant resistance to pathogens. *P. ogarae* F113 possesses many plant-beneficial functions that promote plant growth.^15^^,^^36^^,^^37^ Castro and colleagues (2019)^37^ showed that *P. putida* and *P. fluorescens* stimulate the growth of *A. thaliana* with an induction of auxin response in root tips. In our study, both F113 *Δcrc* and *ΔcbrB* were unable to stimulate the auxin signaling in Col-0 roots in opposition to F113 WT, indicating a possible role of revCCR in auxin hormone signaling. Auxin present in plant roots during bacterial colonization has a dual origin: PGPR are able to produce auxin perceived by the plant, but they also induce auxin production in plants themselves.^38^ Among auxins, indole-3-acetic acid (IAA) produced by the bacteria is perceived by the plant and modulates the plant’s root structure, as shown by the inoculation of IAA deficient *P. putida* mutant on canola and mungbean roots.^39^^,^^40^ IAA produced by *Rhizobium* increases nodule number on *Medicago* roots.^41^ The lack of auxin signaling induction in *Arabidopsis* roots by the F113 *Δcrc* and *ΔcbrB* mutants (Figures 6E and 6F) indicates that revCCR is probably implicated in plant auxin induction and thus plant growth promotion. On the other hand, we demonstrated that bacterial root colonization capacity is dependent on revCCR, as root colonization is hampered in F113 *Δcrc* and *ΔcbrB* (Figures 6A and 6B). Thus, the lack of auxin response after inoculation by F113 *Δcrc* and *ΔcbrB* might be due to inefficient root colonization.

In addition to stimulating plant growth, another important aspect of PGPR beneficial actions on plants is the induction of systemic resistance (ISR).^22^ Although the underlying mechanisms involved are still not fully understood, JA is indicated as a key player at several levels. JA is implicated in the early steps of plant-microbe interaction by suppressing SA dependent defense for successful colonization by PGPR, as well as at later stages for plant defense priming but also in response in case of the attack against the aerial part of plants.^42^ Our results show that both *P. ogarae* F113 WT and mutants *Δcrc* and *ΔcbrB* are able to induce similar levels of JA response in inoculated Arabidopsis roots (see Figure S2) indicating that rev CCR is not implicated in inducing plant defense. The bacterial mechanisms that ignite the induction of ISR in plants are still to discover.

**Figure 7:**
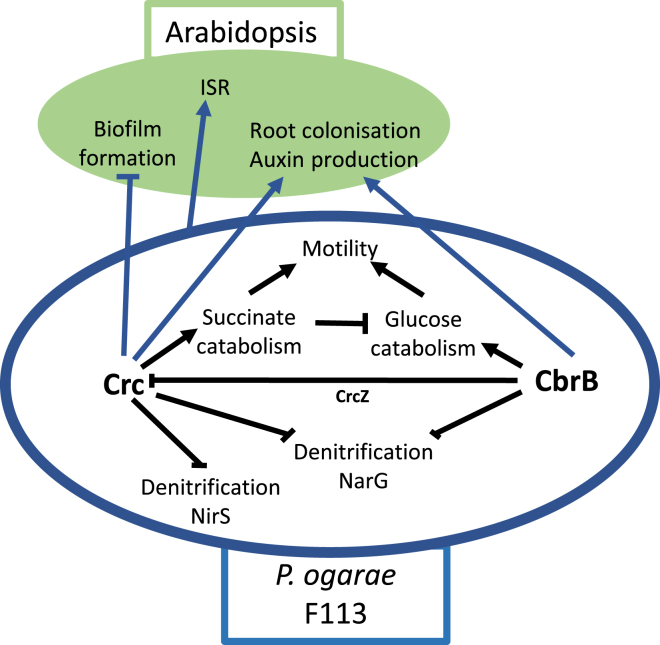
Proposed model of C and N transformation and bacterial traits regulation using Carbon Catabolic Repression (rev-CCR) mechanism

It has been described that plant species are able to tailor their microbial communities in the rhizosphere to their need.^3^^,^^43^ It is well documented that this is achieved by a chemical communication between plants and microorganisms present in the nearby soil. The plant secretes small molecules to the rhizosphere, which are perceived by the microbial community, which in turn modulates root colonization patterns, depending on the perceived signal(s). Our results demonstrate that bacterial carbon source preference and its major regulators Crc and CbrB are involved in this communication between the plant *Arabidopsis* and the bacteria *P. ogarae* F113, shaping bacterial response to plant signals, and thus CCR plays a major role in bacterial root colonization. Importantly, our study highlights that different bacterial molecular mechanisms are involved in responding to plant signals, some of them depending on preferential carbon source use, such as biofilm formation, while others are independent of CCR and are probably more general responses to PGPR colonization, as the modification of JA signaling and likely ISR induction (Figure 7).

In conclusion, as highlighted by Haichar et al. (2014),^11^ depending on environmental conditions, the composition of exuded carbon sources shifts. This influences the functional traits expressed by the plant-associated microbiota via the CCR mechanisms. As already suggested by Franzino et al. (2022),^8^ the interaction between compatible CCR bacteria (e.g., *Bacilus*) and rev-CCR bacteria (e.g., *Pseudomonas*) in the rhizosphere may increase beneficial effects on plant growth and health through the cooperative allocation of plant-released compounds. In the case of rev-CCR strains, genes encoding bacterial traits involved in plant growth and health could be up or down regulated by rev-CCR according to plant root exudates composition in carbon sources. Compatibility between rev-CCR mechanisms and plant root exudates could maximize bacterial potential to promote plant health and growth.

### Limitations of the study

Catabolic carbon repression is a complex system affecting numerous metabolic pathways through a titration mechanism via ncRNA. Future works should focus on interplay between these ncRNAs and other regulators such as ANR talked in this study.

## Resource availability

### Lead contact

Requests for further information and resources sould be directed to and will be fulfilled by the lead contact, Feth el Zahar Haichar (feteh-el-zahare.haichar@insa-lyon.fr).

### Materials availability

All material reported in this article will be shared by the lead contact upon request.

### Data and code availability

All study data are included in the article or the Supporting Information. All data reported in this article will be shared by the lead contact upon request. No code was used in this study.

## Acknowledgments

This work was supported by financial supports from the 10.13039/501100001665French National Research Agency (ANR-18-CE32-0005, DIORE) and from National Institute of Applied Sciences, INSA Lyon to FZH and from the French “Institut National de Recherche pour l'Agriculture, l'Alimentation et l'Environnement. (10.13039/501100022077INRAE) and from Ecole Normale Supérieure-Lyon, France to JS and MB. We thank Patrice Bolland and Alexis Lacroix (ENS de Lyon France) and Elise Lacroix (University Lyon 1, FR BioEnviS) for plant handling. We gratefully acknowledge the PLATIM imaging platform of the IFR BioScience Lyon (UMS3444/US8) for confocal microscopy experiments. We also thank Erwan Guguen from MAP laboratory for genetic construction advises.

## Author contributions

FZH designed the research. FZH, HB, JS, MB, and TF planned the experiments. TF, LM, AM, JS, MD, YML, and FMB performed and analyzed experiments. TF, JS, and FZH wrote the article. All the authors edited the article.

## Declaration of interests

The authors declare no competing interests.

## STAR★Methods

### Key resources table

**Table:** 

REAGENT or RESOURCE	SOURCE	IDENTIFIER
**Bacterial and virus strains**		

*Pseudomonas ogarae* F113	Shanahan et al.^14^	GCA_000237065.1
*P. ogarae* F113 *Δcrc*	This study	N/A
*P. ogarae* F113 *Δcbrb*	This study	N/A
For detailed strains used in study for mutant construction please see Table S1	NA	NA

**Critical commercial assays**		

TAKARA PrimeScript™ RT Reagent Kit	Takara Bio Inc	Cat. # RR092A
TAKARA TB Green Premix Ex Taq™ (Tli RNaseH Plus) Kit	Takara Bio Inc	Cat. # RR420A

**Experimental models: Organisms/strains**		

*Arabidopsis thaliana*	The European Arabidopsis Stock Center	N1092
*A. thaliana*: DR5::mTurquoise	Galvan-Ampudia et al.^44^	N/A
*A. thaliana*: p35S::JAZ1-Gus	Thines et al.^45^	N/A

**Oligonucleotides**		

For primer sequences, please see Table S2	NA	NA

**Software and algorithms**		

MassLynx 4.1	Waters	https://www.waters.com
ImageJ	Public domain	https://imagej.net/ij/

**Other**		

CFX Connect Real-Time PCR	Bio-rad	N/A

### Experimental model and study participant details

This study used the rhizobacteria Pseudomonas ogarae F113 isolated from sugar beet (also known as P. fluorescent F113) and the plant *Arabidopsis thaliana* Columbia-0 obtained from the european arabidopsis stock center (stock number N1092).

### Method details

#### Bacterial strains and growth conditions

Bacterial strains and the plasmids used in this study are listed in Table S1. Bacteria were grown in Lysogeny Broth (LB) or LB plate (1.5% agar) at 30°C for *P. ogarae* F113 or 37°C for *Escherichia coli*. When required, antibiotics were added to the media at the following final concentration: 100 μg mL^−1^ for ampicillin, 10 μg mL^−1^ for tetracycline, 25 μg mL^−1^ for kanamycin and 15 μg mL^−1^ for gentamycin.

#### Construction of *P. ogarae* F113 mutants

The primers used in this study are listed in Table S2.

##### F113 *Δcrc*

The Cre/Lox recombinase strategy^46^ was used to construct *P. ogarae* F113 Δ*crc* mutant. Briefly, upstream and downstream region of *crc* were amplified by PCR (≈1 kb) using pCM184-UPcrcF113 and pCM184-DOWNcrcF113 primers (Table S2). Both purified products were then cloned into pCM184 vector using ABM pro ligation free cloning kit (Applied Biological Materials Inc., Canada) between the restriction enzyme sites BglII/NotI and ApaI/SacI, respectively. The constructed plasmid was transferred into *P. ogarae* F113 strain by biparental mating using *E. coli* MFDlpir strain.^47^ Two crossing-over events allowed to remove the plasmid (tetracycline resistance) and the *crc* gene. Positives clones were then screened on agar plate supplemented with kanamycin (Km) or tetracycline (Tc), looking for Km^R^ and Tc^S^ clones. After that, the pCM157 containing the *cre* recombinase gene was introduced by biparental mating to induce the recombination of the two *LoxP* sites, excising the kanamycin resistance gene, in order to finally obtain a mutant with a disruption of *crc* gene and without any resistance gene added. Kan^S^ and Tc^S^ clones were screened for mutated *crc* gene by PCR and Sanger sequencing (Microsynth, France).

##### F113 *ΔcbrB*

The suicide vector strategy by pk18mobsacB vector^48^ was used to construct a *P. ogarae* F113 *ΔcbrB* mutant. Two regions, upstream and downstream of *cbrB,* were PCR amplified (≈1 kb) using pK18-UPcbrBF113 and pK18-DOWNcbrBF113 primers (Table S2), respectively, and assembled by PCR overlap to form a 2 kb product containing a deleted version of *cbrB*. The purified product was then cloned by recombination into linearized pK18*mobsacB* ABM pro-ligation free cloning kit (Applied Biological Materials Inc., Canada). The cloned region was verified by Sanger sequencing (Microsynth, France). Then, the plasmid construct was introduced into *P. ogarae* F113 strain by biparental mating using *E. coli* MFDlpir strain. Simple crossing over between homologous regions present in this non-replicative plasmid allowed its integration into the targeted site of the genome (upstream or downstream region of *cbrB* gene). Thus, transconjugants were screened by using one primer outside the region (F113cbrBfv or F113cbrBrv) and one primer inside the plasmid (M13fw or M13rv). The second crossing event will remove the plasmid (the part containing *sacB* and kanamycin resistance) with or without the *cbrB* gene. Positives clones were then screened on agar plate supplemented with sucrose 15% with or without kanamycin. Clones Km^S^ and Suc^R^ were then selected and the correct excision of *cbrB* gene was then validated by Sanger sequencing (Microsynth).

##### Complementation

To perform complementation of the two mutant strains F113 Δ*crc* and F113 Δ*cbrB*, *crc* and *cbrB* genes with their promoter region (200 nucleotides before starting codon) were amplified by PCR (pBBR1-CompcrcF113 and pBBR1-CompcbrBF113 primers, Table S2) and then cloned by ligation into pBBR1-MCS5 vector (Table S1) using ApaI/SacI sites. The two cloned regions were sequenced. Then the constructed vectors were transferred in *P. ogarae* F113 mutant strains using electroporation and selected on LB plate supplemented with 15 μg mL^−1^ gentamycin.

#### F113 kinetic growth conditions

*P. ogarae* F113 and its derived mutants were grown at 30°C in 5 mL of M9 minimal salt (Difco, USA) supplemented with 40 mM of carbon from either glucose or succinate. Under denitrification conditions, 20 mM of KNO_3_ were added and helium was injected into sterile tubes in order to generate anoxic conditions, and cells were grown without shaking. Overnight cultures were harvested and washed with a 0.9% NaCl solution and each strain was inoculated in each medium condition at OD_600nm_ 0.075. Growth was then monitored each 2 h for aerobic condition and each 24 h for anaerobic condition using an Ultrospec 10 Cell density meter (Amersham Biosciences, UK). For aerobic condition, the growth rate (μ) was calculated during the linear exponential phase; μ = (log2*N2* − log2*N1*)/(*t2* − *t1*) where *N2* and *N1* are the OD measured at times *t2* and *t1*, respectively. Each condition was performed in triplicates and this experiment was repeated in three independent times.

#### Carbon (C) and nitrogen (N) quantification

One mL of bacterial culture sample was collected from each tube during growth under anaerobic conditions and filtered using a 0.2 μm filter to remove bacterial cells from the medium. Succinate^49^ and glucose^50^^,^^51^ in the samples were quantified by HPLC. Under our conditions, succinate was eluted at a retention time of 7 min and glucose at 8.5 min. Quantification was performed by measuring peak areas using the MassLynx 4.1 software (Waters, France) and compared with a standard curve of succinate or glucose to quantify the amount of each compound in each sample.

For N source quantification, N–NO_3_^-^ concentrations in samples were determined by cadmium reduction of nitrate and colorimetric determination of produced nitrite^52^ using a sequential analyzer (SmartChem200, AMS Alliance, Frépillon, France).

#### Biofilm formation

Biofilm forming ability of *P. ogarae* F113 wild-type and mutant strains on abiotic surface was assayed according to Sivakumar et al. (2020).^5^ Briefly, overnight cultures were washed with NaCl 0.9% and resuspended in K10T-1 medium^53^ at OD_600nm_ of 0.05. Two-mL samples were dispensed into glass tubes in 4 replicas. The sterile medium served as a negative control. Tubes were incubated at 30°C without shaking. After 24 h, the medium with planktonic cells was discarded, tubes were washed with NaCl 0.9%, and the biofilm was stained with 2.5 mL of crystal violet (0.1% w/v) for 20 min. The dye was removed, the tubes were washed with sterile water, air-dried and photographs were then taken. In order to measure biofilm formation, the produced biofilm was eluted with 95% ethanol by shaking tubes for 20 min to ensure the crystal violet dissolution. Then, 200 μL was transferred into a 96-well flat-bottom plate and the absorbance was measured at 590 nm on a TECAN Spark.

#### Motility assays

Overnight cultures were washed with sterile saline buffer (0.9% NaCl) and diluted to OD_600nm_ = 0.1. Five μL were stabbed at the center of minimal medium M9 plate containing 0.3% agar and supplemented with 40 mM of carbon from either glucose or succinate in 4 replicates for each strain. The swimming zone was measured after 120 h at 30°C. *Dickeya dadantii* was used as a classic CCR swimming control strain.^54^

#### *In vitro* competition assays

A culture of only one strain (F113 WT, F113 Δ*crc* or F113 Δ*cbrB*) or a co-culture of the wild-type strain and a mutant strain (F113 WT and F113 Δ*crc*, or F113 WT and F113 Δ*cbrB*) containing a plasmid with constitutively expressed fluorescent protein (pmRFP or pGFP) at a 1:1 ratio was grown in minimal medium M9 supplemented with either succinate or glucose (40 mM) as sole carbon source. After 24 h of growth, the absolute number of colony-forming units (CFUs) of WT and mutant strains were counted by plating these bacterial suspensions on LB supplemented with 25 μg mL^−1^ of kanamycin or spectinomycin. The colonies, displaying red or green coloration according to the harbored plasmid, were screened on a blue-light transilluminator. The two combinations of reporter plasmids were tested to verify that they had no significant effect on bacterial fitness.

#### Plant growth and analyses

Seeds of *Arabidopsis thaliana* accession Columbia-0 (Col-0) were surface-sterilized by vapor-phase according to Clough and Bent (1998), sown on plates containing Murashige and Skoog (MS) (Duchefa, The Netherland) medium supplemented with 0.8% plant agar and then grown under a 16 h light/8 h dark condition at 22°C in a growth chamber.

#### *In planta* competition assay

Seven days-old *A. thaliana* Col-0 seedlings were transferred to six-well culture plates. Each well contained 6 mL of liquid MS (No carbon sources were added) and was inoculated with 100 μL (at 0.1 DO) of a single strain culture (F113 WT, F113 *Δcrc* or F113 *ΔcbrB*) or a co-culture of two strains (F113 WT and F113 *Δcrc* or F113 *ΔcbrB*) containing a plasmid with constitutively expressed fluorescent protein (pmRFP or pGFP) at a ratio of 1:1. Seedlings were then incubated in a growth chamber under 16 h light at 25 °C/8 h dark at 21 °C conditions. After 24 h, roots and medium were vortexed for 5 min to recover bacterial cells, which were then plated on LB solid medium supplemented with 25 μg mL^−1^ kanamycin or 25 μg mL^−1^ spectinomycin. Bacterial enumeration was performed as previously described. The two combinations of reporter plasmids were used to verify that there was no significant effect on bacterial fitness.

#### Effects of *P. ogarae* F113 rev-CCR on plant root architecture

Seven days-old *A. thaliana* Col-0 seedlings were inoculated with 100 μL (at 0.1 DO) of a single strain culture (F113 WT, F113 *Δcrc* or F113 *ΔcbrB*). Seedlings were then incubated in a growth chamber under 16 h light at 25 °C/8 h dark at 21 °C conditions. After 24 h, root architecture of each plant was scanned (Epson Perfection V700 PHOTO, Nagano, Japan) and analyzed with ImageJ software to determine root length and number of secondary roots.

#### Gene expression during root colonization

Three biological repetitions of five 7 days old seedlings (*n* = 5) from Col-0 were transferred into sterile magenta pot containing 30 mL of liquid MS supplemented with 1% sucrose, and then incubated in a growth chamber under a 16 h light at 25 °C/8 h dark at 21 °C conditions. When plants were 14 days old, they were washed in sterile water to remove traces of sucrose and transferred to a new sterile pot containing fresh MS liquid media without sucrose. At 18 days, a suspension of 5.10^6^ cells of *P. ogarae* F113 wild type, F113 *Δcrc* or F113 *ΔcbrB* mutants were inoculated in each pot. Three days after the inoculation, 3 mL of RNA protect (QIAGEN, Germany) were added to each pot. Root associated bacteria were collected by vortexing the root system with 1 mL of medium. Culture medium was collected, and bacteria were pelleted by centrifugation at 7300 rpm at 4 °C. Bacteria were freeze-dried in liquid nitrogen and conserved at −80 °C until RNA extraction.

Total RNA from pellets were extracted using RNeasy Plus Mini Kit (QIAGEN, Germany). DNA traces were eliminated with DNase I treatments (NEB, USA). Isolated RNA was quantified using Nanodrop ND-1000 spectrophotometer (Labtech, Italy), and 500 ng of total DNA-free RNA was reverse transcribed using PrimeScript RT Reagent Kit (TAKARA, Japan). qRT-PCR was performed using the TB Green Premix Ex Taq (Tli RNase H Plus) kit (TAKARA, Japan). CFX Connect Real-Time PCR detection system from BIO-RAD was used for thermal cycling reactions according to the following protocol: an initial step at 95°C for 10 min, followed by 40 cycles at 95°C for 30 s, 60°C for 30 s and 72°C for 30 s and a final step at 72°C for 3 min in order to amplify *crcZ, crcY* and denitrification (*narG, nirS, norB, nosZ*) genes (Table S2). Normalization of gene expression was performed using *rpoD* housekeeping gene. Fold change was calculated with the ΔΔCt method according to Livak and Schmittgen (2023).^55^ Each condition was performed in triplicate.

#### Bacterial effect on plant hormone accumulation

Ten days old *A. thaliana* seedlings harboring the *DR5::mTurquoise* auxin reporter^44^ or the p35S::JAZ1-Gus jamonate reporter^45^ were inoculated with 1.10^6^ cells of *P. ogarae* F113 wild type, F113 *Δcrc* or F113 *ΔcbrB* mutants in 100 μL of 0.9% NaCl suspension. Those suspensions were spread along the root systems, which were incubated in a growth chamber under a 25°C, 16 h light/21°C 8 h dark condition for 24 h prior to observations.

For auxin signaling, 20 root apexes by plant conditions were imaged by using a Zeiss LSM700 laser confocal scanning microscope, according to Brunoud et al. (2020).^56^ For jasmonate signaling, roots were fixed and stained for GUS activity^57^ (Béziat et al., 2017). Stained roots were imaged with a Leica upright binocular microscope. Integrated fluorescence density of GUS coloration was quantified by ImageJ software.

### Quantification and statistical analysis

Kinetic growth and expression profiles were performed in triplicate, other experimentations in quadruplicate. Unless specified, Mann-Whitney’s U-test was applied to determine whether significant difference in growth was observed between conditions (the two carbon sources for each time, strain and aerobic/anaerobic conditions). Unless specified, stars indicate a *p*-value <0.05.
