# Supplementary material for: Carbon source utilization regulates biofilm formation and plant-beneficial interactions of Pseudomonas ogarae F113
Source: iScience. 2025 Sep 24;28(11):113639. doi: 10.1016/j.isci.2025.113639 (PMC12552145; doi:10.1016/j.isci.2025.113639)
Supplement: Document S1. Figures S1 and S2 and Tables S1 and S2 [file mmc1.pdf]

**iScience, Volume *28***

## Supplemental information

# Carbon source utilization regulates biofilm formation and plant-beneficial interactions of *Pseudomonas ogarae* F113

**Théophile Franzino, Hasna Boubakri, Lisa Merlin, Amandine M'Sakni, Michel Droux, Florian Mermillod-Blondin, Yvan Moënne-Loccoz, Mohammed Bendahmane, Judit Szécsi, and Feth el Zahar Haichar**

**Table S1. Strains and plasmids used in this study**

| Strains | Genotype/relevant features | Source/reference |
|---|---|---|
| ***P. ogarae*** | | |
| F113 | Wild type strain | Shanahan et al., 1992 |
| F113 pBBR1-MCS5 | F113 wt harborring pBBR1-MCS5 empty | This study |
| F113 Δ*crc* | F113 deleted of *crc* gene | This study |
| F113 Δ*crc* pBBR1-MCS5 | F113 Δ*crc* harborring pBBR1-MCS5 empty | This study |
| F113 Δ*crc-crc* | F113 Δ*crc* harborring pBBR1-MCS5::*crc* F113 | This study |
| F113 Δ*cbrB* | F113 deleted of *cbrB* gene | This study |
| F113 Δ*cbrB* pBBR1-MCS5 | F113 Δ*cbrB* harborring pBBR1-MCS5 empty | This study |
| F113 Δ*cbrB-cbrB* | F113 Δ*cbrB* harborring pBBR1-MCS5::*cbrB* F113 | This study |
| | | |
| ***E. coli*** | | |
| DH5α | $F^-$ *endA1 glnV44 thi-1 recA1 relA1 gyrA96 deoR nupG purB20* ϕ80d*lacZ*ΔM15 Δ(*lacZYA-argF*)U169, hsdR17($r_K^-m_K^+$), λ– | Grant et al., 1990 |
| MFDλpir | MG1655 RP4-2-Tc::[ΔMu1::aac(3)IV-ΔaphA-Δnic35-ΔMu2::zeo] ΔdapA::(erm-pir) ΔrecA | Ferrières et al., 2010 |
| | | |
| **Plasmids** | | |
| pCM157 | Vector containing Cre recombinase activity, $Amp^R$, $Tc^R$ | Marx and Lidstrom 2002 |
| pCM184 | Cloning vector used for homologous recombination, $Amp^R$, $Km^R$, $Tc^R$ | Marx and Lidstrom 2002 |
| pCM184::Up*crc*F113 | pCM184 carrying Upstream sequence of F113 *crc* gene | This study |
| pCM184::UD*crc*F113 | pCM184::UpcrcF113 carrying Downstream sequence of F113 *crc* gene | This study |
| pK18mobsacB | Cloning vector for allelic exchange; $Km^R$ | Schäfer et al., 1994 |
| pK18mobsacB::*cbrB*F113 | pK18mobsacB carrying F113 *cbrB* sequence cloned into pK18mobsacB, | This study |
| pBBR1-MCS5 | pBBR1-MCS5 stable vector; $Gm^R$ | Kovach et al., 1995 |
| pBBR1-MCS5::*crc* F113 | pBBR1-MCS5 carrying F113 *crc* gene with promoting region sequence cloned into pBBRMCS5 | This study |
| pBBR1-MCS5::*cbrB* F113 | pBBR1-MCS5 carrying F113 *cbrB* gene with promoting region sequence cloned into pBBRMCS5 | This study |
| pBAV1K-T5-gfp | T5lacO; KanR | Bryksin and Matsumura, 2010 |

## Table S2. Primers used in this study

| Name | Sequences (5'->3') | Restriction site | Target | Type of utilization | Product Size |
|---|---|---|---|---|---|
| pCM184-UPcrcF113fw | CCCGAAAAGTGCCACCTGACGTCTAGATCTAGATCTTCACCGACACTGGC | BglII | Upstream region of *crc* | Deletion of *crc* | 1kb |
| pCM184-UPcrcF113rv | tcgtataatgtatgctatacgaagttatgcggccgCATGATCCGCATAAATGGCCCC | NotI | | | |
| pCM184-DOWNcrcF113fw | gaagttatggatccagcttatcgatacccgggCGTACCGGCGACATCC | ApaI | Downstream region of *crc* | | 1kb |
| pCM184-DOWNcrcF113rv | tcggctggatcctctagtgagctcGTTGATGGGATTGCTGACCG | SacI | | | |
| pK18-UPcbrBF113fw | aggaaacagctatgacatgattacgaattcaaggcttgctgcaaggtttcatcg | EcoRI | Upstream region of *cbrB* | Deletion of *cbrB* | 1kb |
| pK18-UPcbrBF113rv | cggtgttacccccacacctcagctgatcaaaatgtgcggcattgattcg | | | | |
| pK18-DOWNcbrBF113rv | cgaatcaatgccgcacattttgatcagctgaggtgtgggggtaacaccg | | Downstream region of *cbrB* | | 1kb |
| pK18-DOWNcbrBF113rv | cctgcaggtcgactctagaggatccccggggcgctggtggcgacgtcgaaatcc | SmaI | | | |
| pBBR1-CompcrcF113fw | GGGCCCGGGCCCcggaaccggtgttgaacaggc | ApaI | F113 crc gene and promoter | Complementation of *crc* | 1kb |
| pBBR1-CompcrcF113rv | GAGCTCGAGCTCgcgatacgggcattttctttgg | SacI | | | |
| pBBR1-CompcbrBF113fw | GGGCCCGGGCCCagtgaagccttcgaacacacgg | ApaI | F113 cbrB gene and promoter | Complementation of *cbrB* | 1,7kb |
| pBBR1-CompcbrBF113rv | GAGCTCGAGCTCcggtgttacccccacacctcag | SacI | | | |
| F113cbrBfw | ccgtcgagagaatcgaatcaatgc | | F113 *cbrB* gene | Screen for F113 *cbrB* gene | 1.5kb |
| F113cbrBrv | cggtgttacccccacacctcag | | | | |
| M13fw | CGCCAGGGTTTTCCCAGTCACGAC | | MCS | Screen for plasmid construction | |
| M13rv | TCCTGTGTGAAATTGTTATCCGCT | | | | |
| qPCR F113 rpoD fw | GTCGCGCGAAGAAAGAGATG | | *rpoD* | qPCR *rpoD* | 94pb |
| qPCR F113 rpoD rv | AGATCGAGGAATTGCAGGCC | | | | |
| qPCR F113 narG fw | AAGAACGGCCTGATCACCTG | | *narG* | qPCR *narG* | 157pb |
| qPCR F113 narG rv | GCTTGCGGATTTTCGGGTAC | | | | |
| qPCR F113 nirS fw | TTGTCGTCGATCACCACCAG | | *nirS* | qPCR *nirS* | 121pb |
| qPCR F113 nirS rv | GTATCAAGAAAGGCGCGCTG | | | | |
| qPCR F113 norB1 fw | CTTTGGGTGGAAGGCGTTTG | | *norB* | qPCR *norB* | 152pb |
| qPCR F113 norB1 rv | AGCCACTTCTCGACCACTTC | | | | |
| qPCR F113 nosZ1 fw | CTGGGGAGCTGGACGAATAC | | *nosZ* | qPCR *nosZ* | 101pb |
| qPCR F113 nosZ1 rv | CTTGCTTTCGTTGGTCAGGC | | | | |
| qPCR F113 crcZ fw | AGAGGCGTTGTATTTGGGGC | | *crcZ* | qPCR *crcZ* | 117pb |
| qPCR F113 crcZ rv | TGCTTTGATCGCTGTGTTGC | | | | |
| qPCR F113 crcY fw | TTGCTCTTGATCCCGGATGG | | *crcY* | qPCR *crcY* | 114pb |
| qPCR F113 crcY rv | AATTTTGTCGTCGCGTGCTC | | | | |

**Figure S1. Growth kinetics in aerobic conditions of *Pseudomonas ogarae* F113 WT and its complemented mutants.** Histographic representation of growth of **(A)** F113 WT harboring an empty pBBR1-MCS5, **(B)** F113 *Δcrc* harboring pBBR1-MCS5::*crc* and **(C)** F113 *ΔcbrB* harboring pBBR1-MCS5::*cbrB*. Cells were grown in M9 minimal medium supplemented with 40 mM of carbon either from succinate (black bar) or glucose (grey bar) as sole carbon source in aerobic conditions and OD 600nm was measured during 8 hours. Error bars correspond to standard deviations of three biological replicates. Stars indicate a $p$-value < 0.05 (Mann-Whitney's U-test).

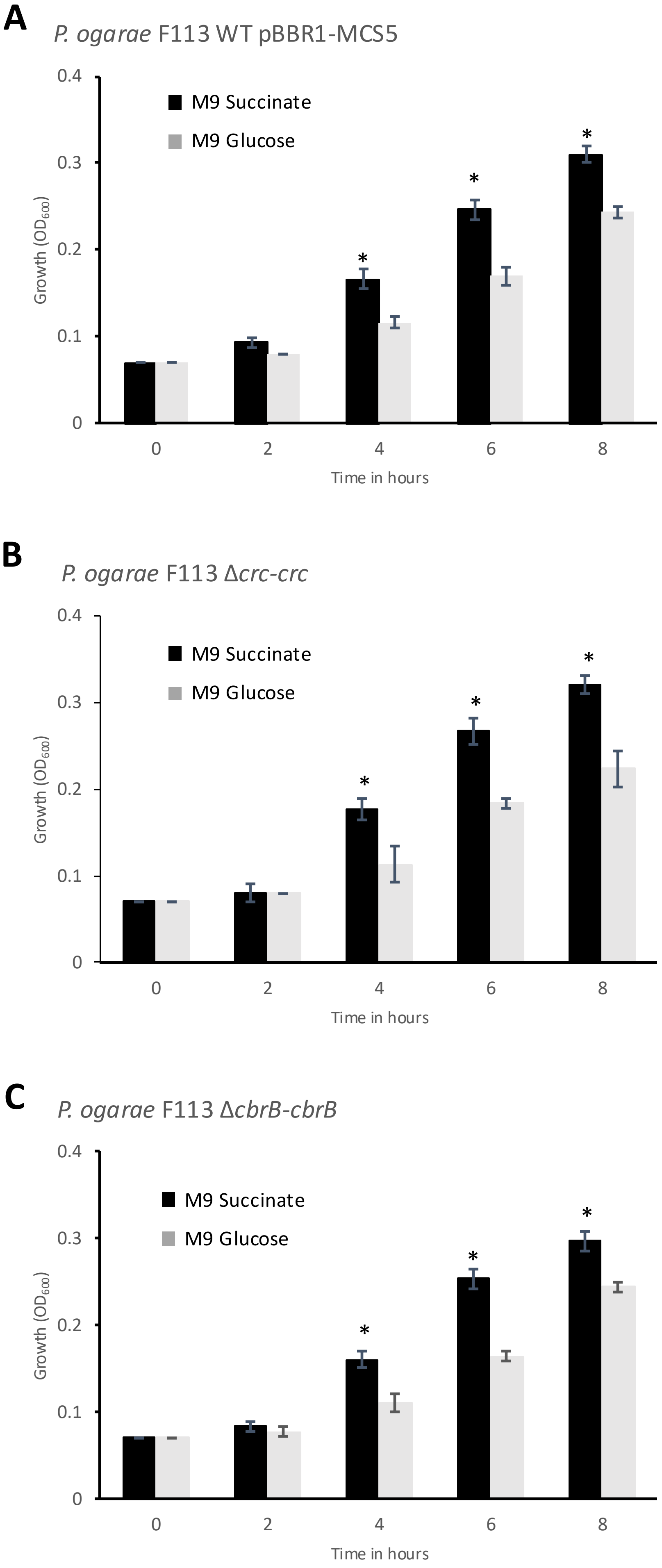
A
P. ogarae F113 WT pBBR1-MCS5
M9 Succinate
M9 Glucose
Growth (OD600)
Time in hours
B
P. ogarae F113 Δcrc-crc
M9 Succinate
M9 Glucose
Growth (OD600)
Time in hours
C
P. ogarae F113 ΔcbrB-cbrB
M9 Succinate
M9 Glucose
Growth (OD600)
Time in hours

**Figure S2. Effect of revCCR on jasmonate signalling.** *A. thaliana* roots were inoculated with F113 WT, F113 Δcrc or F113 ΔcbrB and their effect on jasmonate was determined 24h after inoculation. (A) representative images of roots harbouring the 35S::JAZ1-Gus jasmonate signalling marker after inoculation with F113 WT or mutant strains. Bar represents 1 mm. (B and C) Quantification of Gus integrated intensity in roots expressing 35S::Gus or the 35S::JAZ1-Gus jasmonate signalling marker after inoculation with F113 WT or mutant strains. Error bars correspond to standard errors (n = 8, 9, 9, 7, 11, 13, 11, 13 for (B) and n = 11, 11, 11, 11, 14, 16, 16, 18 for (C)). * $p$-value < 0.1; ** $p$-value < 0.01 (Student's $t$-test).

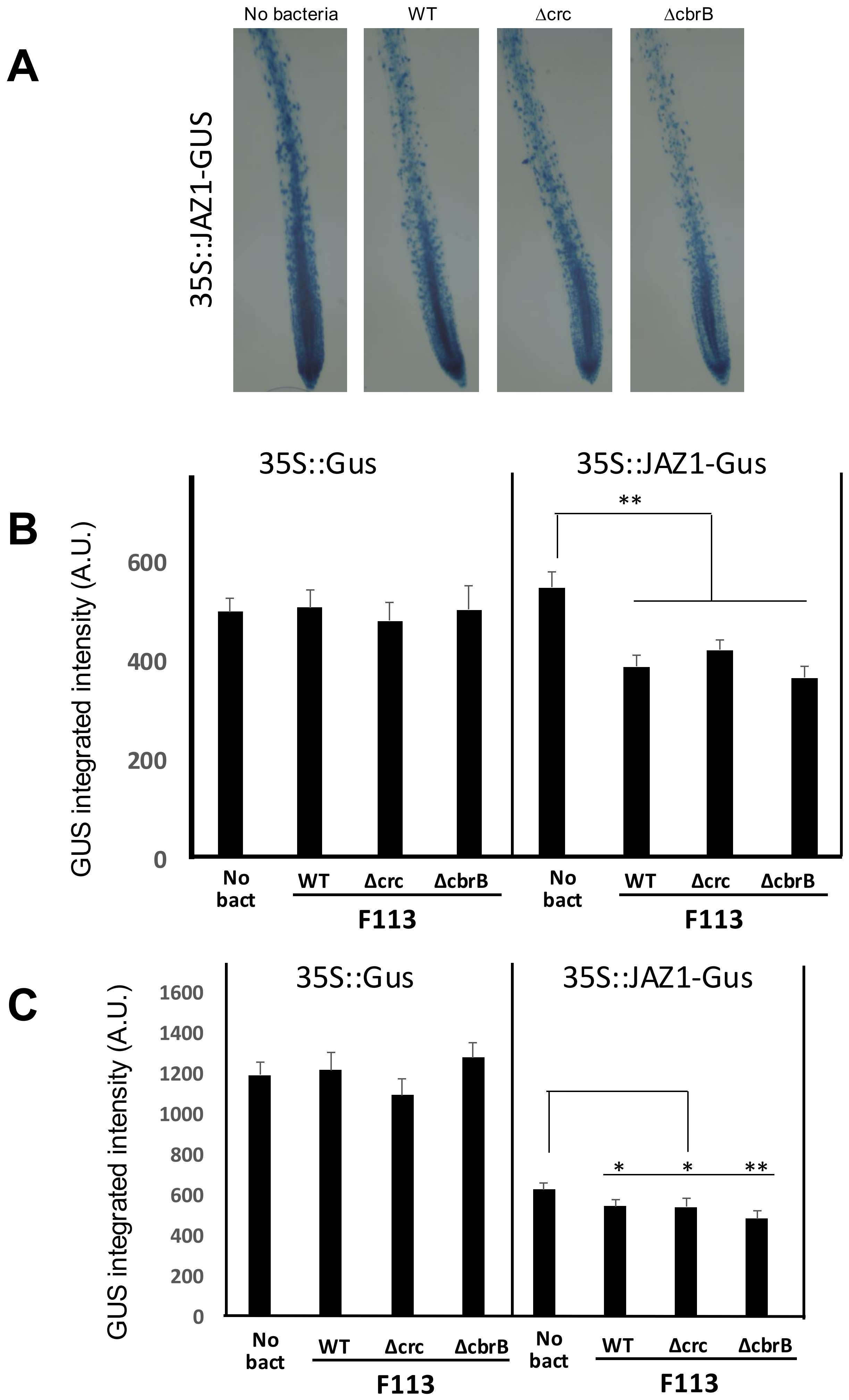

A
No bacteria
WT
Δcrc
ΔcbrB
35S::JAZ1-GUS
B
35S::Gus
35S::JAZ1-Gus
GUS integrated intensity (A.U.)
600
400
200
0
**
No bact
WT
Δcrc
ΔcbrB
F113
C
35S::Gus
35S::JAZ1-Gus
GUS integrated intensity (A.U.)
1600
1400
1200
1000
800
600
400
200
0
*
*
**
No bact
WT
Δcrc
ΔcbrB
F113
